# Introduction to Nanomaterials Applied to Life Sciences

**DOI:** 10.1039/d1na90011k

**Published:** 2021-02-17

**Authors:** A. Espinosa, F. J. Teran, D. Ortega

**Affiliations:** IMDEA Nanoscience Faraday 9 28049 Madrid Spain daniel.ortega@uca.es; Institute of Research and Innovation in Biomedical Sciences of the Province of Cádiz (INiBICA), University of Cádiz 11002 Cádiz Spain; Condensed Matter Physics Department, Faculty of Sciences, University of Cádiz, Campus Universitario Río San Pedro s/n 11510 Puerto Real (Cádiz) Spain

## Abstract

A. Espinosa, F. J. Teran and D. Ortega introduce the *Nanoscale Advances* themed collection on Nanomaterials Applied to Life Sciences.
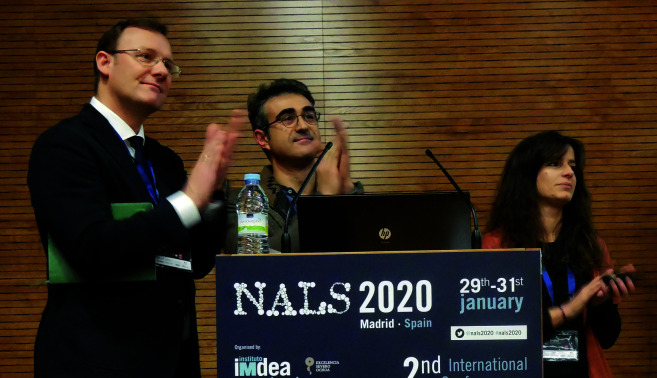

Nanotechnology is providing unprecedented opportunities to develop new functional materials with unique electronic, optical, and magnetic properties with vast potential in many areas. Indeed, an ever-increasing number of life sciences, environmental and energy applications are adopting nanotechnology to a variable extent to go beyond the current state-of-the-art and generate innovative ways of tackling the most pressing needs of today’s society. This situation is driving the advances in the existing manufacturing procedures, which now require operations to be carried out under specifically devised standards for nanomaterials. Some of these standards have been recently released, while many others are still under development.^1,2^ Funding agencies and the relevant industries seek wealth generation from their investments in research and development, and are therefore willing to transform this know-how into quality control processes that will eventually lead to more profitable yields. However, before we open the door to all these innovations, there is a pending need for a thorough assessment of the many health, environmental, standardization and ethical aspects of nanomaterials.

More and more companies worldwide look to exploit the expanding range of novel properties that are being discovered in nanomaterials. How this will be streamlined is yet to be seen. While some nanomaterials have been designed to solve a specific real-life issue, lessons learnt from the past show us that innovations often do not follow this pathway forward. Nanomaterials for a clearly defined indication can be repurposed to multiply their benefits, which is exemplified by the case of iron oxide nanoparticles, which were mainly formulated as iron supplements, but they found applications as magnetic resonance imaging and hyperthermia agents, among others. These cases bear an incalculable practical value, since they represent effective ways of cutting development costs and regulatory approval times, thus accelerating their access to market. In addition, we have witnessed how ‘old-fashioned’ nanomaterials with more than 50 years behind them may become lifeguards when you least expect it. The most recent example is the use of lipid nanoparticles in COVID-19 vaccines to transport mRNA molecules.^3^

Within this framework, the *2*^*nd*^*International Conference on Nanomaterials Applied to Life Sciences 2020* (NALS 2020) was held on 29^th^–31^st^ January 2020 at the Madrid Institute for Advanced Studies in Nanoscience (IMDEA Nanociencia). More than 200 participants, coming from 15 countries got together to establish synergies, foster long-lasting collaborations, and contribute to an academia-industry liaison. The scope of NALS 2020 encompassed synthesis and functionalization of nanomaterials, studies on biocompatibility and toxicity, *in silico* testing, standardisation as well as novel applications in the environment, and for therapy, detection and diagnosis. All these elements were clearly reflected in a gender-balanced program with four invited and twelve keynote speakers with different expertise, working in academic, industrial and clinical sectors. For three days, participants attended 68 talks and 100 poster presentations. Additionally, two round tables were organised—one related to *Women in Science* and the other to *the Value of Scientific and Innovation Networks*—offered dissemination opportunities to students, and exhibited instrumentation technologies in stands set by sponsors.

The present themed collection is the result of this international forum created for presenting and discussing new results around nanomaterials research in the related areas. The selection includes both review and original research articles, covering the most recent progress in the synthesis, characterization, and study of advanced nanomaterials suitable for biomedical applications, such as cancer treatment, immunology and tissue engineering, as well as their degradation and intracellular trafficking pathways. Following is a brief account of the contents, featuring:

- Scaffolds from nanomaterial constructs that can be used to grow muscle skeletal tissue, showing promise for organ-on-a-chip designs, drug screening, transplantation and disease modelling.

- Magneto-plasmonic Janus nanoparticles bearing great potential for diagnostic and therapeutic applications due to the possibility of controlling their self-assembly through multi-stimuli control.

- Magnetic nanoparticles for nanoscale heating that can be used in local thermal therapies and drug-delivery.

- An algorithm for interpreting magnetic characterisation measurements in nanoparticles, aiming for standardisation in nanoparticle size analysis.

- New possibilities in the fields of diagnostics and therapy with microwave-based imaging, sensing and heating of nanomaterials.

- The projection of bioorthogonal chemistry to harness the potential of controlling biological processes by nanostructures with distinct physicochemical properties.

- Pros and cons of using inorganic metal and metal oxide nanoparticles in medicine due to their poorly understood long-term stability and toxicity.

- Immunomodulatory effects of nanomaterials are also presented, with a focus on enhancing their therapeutic potential by modulating the immune response they elicit, as well as their possible leading role in immunotherapy strategies.

- Challenges ahead of our current understanding of the interaction of nanoparticles with biological systems, mainly due to the limited means for isolating and analysing specific intracellular compartments.

There were many other exciting research works that could not be included, but which are still very topical.

As guest editors, we are most grateful to both the contributing authors and the editorial team of *Nanoscale Advances*, in particular for coordinating their efforts to adapt to a publication process that has coincided from the outset with the outbreak and spread of the continuing COVID-19 pandemic.

## Supplementary Material
